# Class switch towards non-inflammatory, spike-specific IgG4 antibodies after repeated SARS-CoV-2 mRNA vaccination

**DOI:** 10.1126/sciimmunol.ade2798

**Published:** 2022-12-22

**Authors:** Pascal Irrgang, Juliane Gerling, Katharina Kocher, Dennis Lapuente, Philipp Steininger, Katharina Habenicht, Monika Wytopil, Stephanie Beileke, Simon Schäfer, Jahn Zhong, George Ssebyatika, Thomas Krey, Valeria Falcone, Christine Schülein, Antonia Sophia Peter, Krystelle Nganou-Makamdop, Hartmut Hengel, Jürgen Held, Christian Bogdan, Klaus Überla, Kilian Schober, Thomas H. Winkler, Matthias Tenbusch

**Affiliations:** ^1^ Institut für klinische und molekulare Virologie, Universitätsklinikum Erlangen und Friedrich-Alexander-Universität (FAU) Erlangen-Nürnberg; Schlossgarten 4, 91054 Erlangen, Germany.; ^2^ Department of Biology, Division of Genetics, Nikolaus-Fiebiger-Center for Molecular Medicine, Friedrich-Alexander-Universität Erlangen-Nürnberg (FAU); Erlangen, Germany.; ^3^ Mikrobiologisches Institut – Klinische Mikrobiologie, Immunologie und Hygiene, Universitätsklinikum Erlangen und Friedrich-Alexander-Universität (FAU) Erlangen-Nürnberg; Wasserturmstr. 3/5, 91054 Erlangen, Germany.; ^4^ Center of Structural and Cell Biology in Medicine, Institute of Biochemistry, University of Luebeck; Luebeck, Germany.; ^5^ Institute of Virology, Freiburg University Medical Center, Faculty of Medicine, University of Freiburg; Freiburg, Germany.; ^6^ Medical Immunology Campus Erlangen, Friedrich-Alexander-Universität (FAU) Erlangen-Nürnberg, Schlossplatz 1, 91054 Erlangen, Germany.

## Abstract

RNA vaccines are efficient preventive measures to combat the SARS-CoV-2 pandemic. High levels of neutralizing SARS-CoV-2-antibodies are an important component of vaccine-induced immunity. Shortly after the initial two mRNA vaccine doses, the IgG response mainly consists of the pro-inflammatory subclasses IgG1 and IgG3. Here, we report that several months after the second vaccination, SARS-CoV-2-specific antibodies were increasingly composed of non-inflammatory IgG4, which were further boosted by a third mRNA vaccination and/or SARS-CoV-2 variant breakthrough infections. IgG4 antibodies among all spike-specific IgG antibodies rose on average from 0.04% shortly after the second vaccination to 19.27% late after the third vaccination. This induction of IgG4 antibodies was not observed after homologous or heterologous SARS-CoV-2 vaccination with adenoviral vectors. Single-cell sequencing and flow cytometry revealed substantial frequencies of IgG4-switched B cells within the spike-binding memory B-cell population (median 14.4%; interquartile range (IQR) 6.7–18.1%) compared to the overall memory B-cell repertoire (median 1.3%; IQR 0.9–2.2%) after three immunizations. Importantly, this class switch was associated with a reduced capacity of the spike-specific antibodies to mediate antibody-dependent cellular phagocytosis and complement deposition. Since Fc-mediated effector functions are critical for antiviral immunity, these findings may have consequences for the choice and timing of vaccination regimens using mRNA vaccines, including future booster immunizations against SARS-CoV-2.

## Teaser

Repeated SARS-CoV-2 mRNA vaccinations shift the antibody response towards IgG4 subclass with decreased FcγR-dependent effector activity.

## INTRODUCTION

During the ongoing pandemic of the severe acute respiratory syndrome coronavirus 2 (SARS-CoV-2) that has reached over half a billion cases worldwide, new and efficient vaccines were developed with unprecedented speed and likely prevented millions of deaths *(**1*, *2**)*. Two mRNA vaccines (Comirnaty from BioNTech/Pfizer and Spikevax from Moderna) were the first mRNA vaccines approved for use in humans. Both showed high efficacies of around 90% in preventing SARS-CoV-2 infections in clinical trials *(**3*, *4**)* and real-world scenarios *(**5*–*8**)*. Several studies documented that antibody responses after a third immunization were superior with regard to their neutralizing capacity against a broad spectrum of SARS-CoV-2 variants of concern (VOC) compared to antibody responses measured after the initial two-dose regimen *(**9*–*11**)*. It was further shown that antibody avidity increased following mRNA booster vaccination, which was partly explained by prolonged germinal center (GC) activation and ongoing B-cell maturation. SARS-CoV-2 vaccine-derived mRNA and spike protein was detected even several weeks after vaccination *(**12**)*. Sequencing of memory B cells revealed somatic hypermutation (SHM) in GCs for up to six months, which resulted in broadening and diversification of the memory B cell repertoire as well as in an improved effectiveness against VOC *(**10*, *12*–*17**)*.

**Fig. 1.**
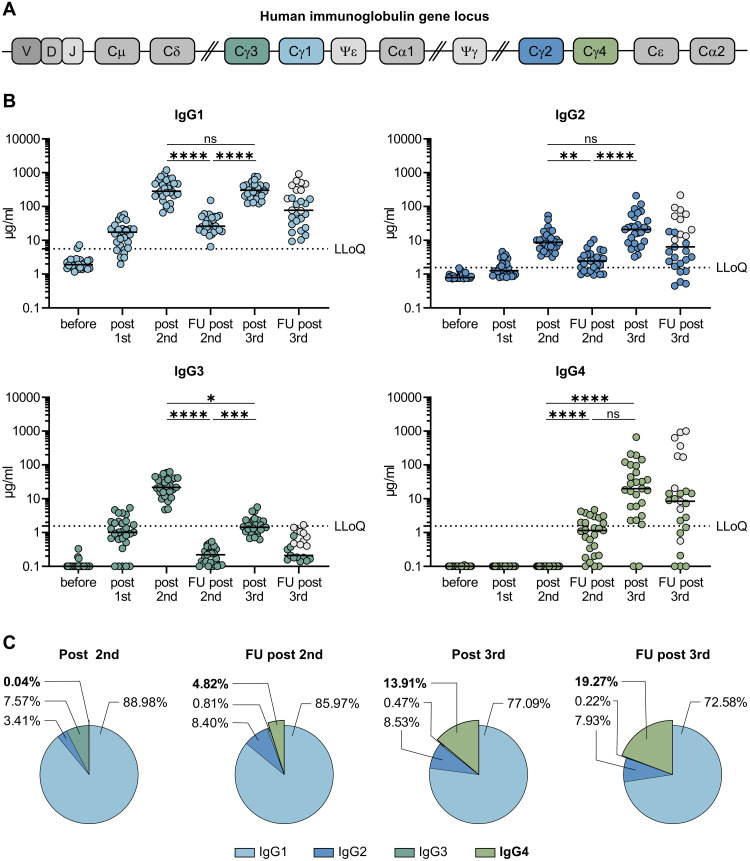
Longitudinal analyses of vaccine induced antibody response. (**A**) Schematic representation of the human immunoglobulin heavy chain gene locus. 5′ of each functional C-region (except Cδ) switch (S) regions are positioned directing class-switch recombination. Gene segments denoted by ψ resemble pseudogenes (**B**) 29 volunteers received three doses of the mRNA vaccine Comirnaty as detailed in Table 1. Serum samples were collected at a median of ten days after each vaccination (post first, post second, post third) as well as during follow-up visits at 210 days after the second vaccination (FU second) and 180 days after the third vaccination (FU third). Ten individuals experienced a breakthrough infection in the time frame between post third and FU third (indicated by grey circles). The different IgG subclasses were quantified by flow cytometry using recombinant monoclonal receptor binding domain (RBD)-antibodies as a standard. The median MFI of three negative sera were used to set the background for each subclass. For visualization purposes, all sera with MFI values below the background were set to 0.1 μg/ml. The lowest limit of quantification (LLoQ) is indicated in each graph by a dotted line and represents the lowest amount of the respective standard mAbs, which was detected (1.56 μg/ml for IgG2, IgG3 and IgG4; 5.6 μg/ml for IgG1). Depicted are individual donors and the respective median. n.a. = not analyzed. For the sake of clarity only statistical comparisons between post second, FU post second, and post third are shown irrespective of statistical significance. (**C**) The proportion of the different IgG subclasses of the total anti-S IgG response is shown for the four last time points. Depicted are the means of each IgG subclass. Over-time comparison within one group was done by Kruskal-Wallis test followed by Dunn’s multiple comparisons test. *p < 0.05, **p < 0.01, ***p < 0.001, ****p < 0.0001, and n.s. indicates not significant.

Activation-induced cytidine deaminase (AID) is the enzyme that catalyzes SHM in antibody variable (V) regions. It is expressed in GC B cells and also mediates class switch recombination (CSR) of constant (C) region genes *(**18*, *19**)*. IgG3 encoded by the γ3 C-region is the most upstream (5′) Cγ-region in the immunoglobulin heavy chain gene locus on chromosome 14 (Fig. 1A). Ongoing activity of AID can lead to switching towards more downstream Cγ regions, i.e. γ1, γ2 and γ4, which encode IgG1, IgG2 and IgG4 *(**20*, *21**)*. CSR is highly regulated during an immune response. The C-region to which a B cell switches is modulated by cytokines and B-cell activators at the level of transcription of non-rearranged heavy chain constant genes *(**21**)*. However, the regulators for germline transcription of the γ2 and γ4 gene locus are not very well understood in humans. IL-4 in concert with IL-10 has been described to be involved in switching to IgG4 *(**22**)*. Most notably, distal IgG variants, in particular IgG2 and IgG4, were reported to mediate mostly non-inflammatory or even anti-inflammatory functions due to decreased Fc-mediated antibody effector functions including antibody-dependent cellular phagocytosis (ADCP), cellular cytotoxicity (ADCC) and complement deposition (ADCD) *(**20**)*.

Shortly after the administration of two doses of SARS-CoV-2- mRNA vaccine (either Comirnaty or mRNA-1273), IgG1 and IgG3 were found to be the predominant IgG subclasses, whereas IgG2 responses were rare and IgG4 responses almost undetectable *(**23*, *24**)*. However, the longitudinal evolution of all four IgG subclasses (IgG1, 2, 3 and 4) in response to mRNA vaccination – and particularly their long-term development after the second and the third dose – has not yet been analyzed.

Here, we report on the analysis of two independent cohorts of vaccinated health-care workers, who developed an increase in anti-spike IgG4 antibodies and IgG4-switched memory B cells five to seven months after the second mRNA immunization with Comirnaty. This response was further boosted by a third mRNA vaccination and/or by breakthrough infections with SARS-CoV-2 VOC. While we confirmed an increased antibody avidity and higher neutralization capacity against the recently emerged Omicron VOC after the third vaccine dose, the switch towards distal IgG subclasses was accompanied by reduced fragment crystallizable (Fc) gamma receptor (FcγR)-mediated effector functions such as ADCP and ADCD.

## RESULTS

### Longitudinal monitoring of anti-spike antibody subclass responses

In a cohort of 29 health care workers (cohort 1), we analyzed the antibody response after SARS-CoV-2 vaccination with three doses of Comirnaty (Table 1). The first two doses were given at an interval of three to four weeks and a further booster vaccination was applied about seven months after the second immunization. Using a flow cytometry-based antibody assay *(**25**)*, anti-spike IgG responses were measured in sera ten days after each vaccination, 210 days after the second and 180 days after the third dose. In line with earlier reports *(**3*, *4**)*, mRNA immunizations induced robust IgG antibody response in all vaccinees (Fig. S1A). Neutralizing capacity was assessed in a surrogate virus neutralization assay confirming the dynamics of vaccine-induced antibody responses (Fig. S1B).

**Table 1.** Characteristics of study cohorts for longitudinal analyses after vaccination with three doses of Comirnaty

	Cohort 1 (CoVaAdapt)	Cohort 2
Vaccines	BNT162b2 mRNA (3x)	BNT162b2 mRNA (3x)
Number of volunteers	n = 29	N = 38
Age in years, median (IQR) [range]	44 (31–52) [24-59]	39.5 (28–53) [25-62]
Sex, n (%) Female	16 (55%)	27 (71%)
Sex, n (%) Male	13 (45%)	11 (29%)
Time intervals between immunizations in days, median (IQR) [range] first to second: second to third:	23 (23–25) [22-28] 227 (216.5–229) [196–244]	23 (22–28) [14-28] 207 (195–216) [170–251]
Time interval from immunization to blood collection in days, median (IQR) [range] post first: post second: Follow-up post second: post third: Follow-up post third:	10 (9–10.5) [8-13] 10 (10–11) [9-11] 210 (209–210) [195–235] 10 (10–10) [9-12] 189 (189–190) [157–202]	14 (14–14) [7-18] 14 (14–17) [13-28] 165 (154–172) [147–194] 17 (14–23) [13-61]] n.a.

Ten days after two immunizations, anti-spike antibodies of the subclasses IgG1, IgG2 and IgG3 were readily detectable in a multiplexed flow cytometric assay, whereas anti-S IgG4 antibodies were undetectable (Fig. 1B). IgG2 levels were markedly lower than IgG3 and IgG1 levels. Intriguingly, 210 days after the second immunization, the levels of spike-specific IgG4 antibodies exceeded the lower limit of quantification in the sera of about half of the vaccinees. The levels for all other subclasses dropped significantly as expected from the overall anti-S response.

To explore whether the rise in IgG4 antibody levels was specific for the homologous mRNA vaccination regimen used, we analyzed sera from an independent cohort *(**26*, *27**)*, in which we compared the immunogenicity of homologous and heterologous vaccination regimens with Comirnaty and the adenoviral vector-based vaccine ChAdOx1 (AZD1222, Vaxzevria) (see Table S1). Five to six months after the second immunization, spike-specific IgG4 antibodies were again detectable in half of the sera of the BNT-BNT cohort, but only in one of the 51 sera from the two other vaccine cohorts (Fig. S2).

After the third mRNA immunization, the amounts of all IgG subclasses were elevated again and reached levels as measured shortly after the second vaccination in the case of IgG1 and IgG2 (Fig. 1B). IgG3 remained at lower levels compared to the time point shortly after second vaccination. Notably, a marked increase in IgG4 antibody levels was observed after the booster immunization in nearly all vaccinees. Until this time point, none of the participants reported an episode of SARS-CoV-2 infection and we could also not detect anti-nucleoprotein antibodies in any of the serum samples.

To analyze the contribution of IgG4 antibodies to the long-lived antibody pool after the third immunization, additional serum samples were collected after a mean period of 180 days from 27 individuals after the third mRNA vaccination. At this time point, several participants reported anamnestic breakthrough infections, which was in line with nucleoprotein serological testing (Fig. 1B, grey dots). Notably, the relative contribution of IgG4 antibodies to the total pool of anti-S IgG increased over time, arguing against a transient expansion of short-lived plasmablasts after the third immunization (Fig. 1C; Fig. S1C). In four individuals IgG4 even became the most prominent IgG subclass after the third immunization. Specifically in individuals having experienced an additional infection, IgG4 antibodies accounted for 40–80% of all anti-S antibodies (Fig. S1C).

### Longitudinal monitoring of class-switching in SARS-CoV-2-specific memory B cells

**Fig. 2.**
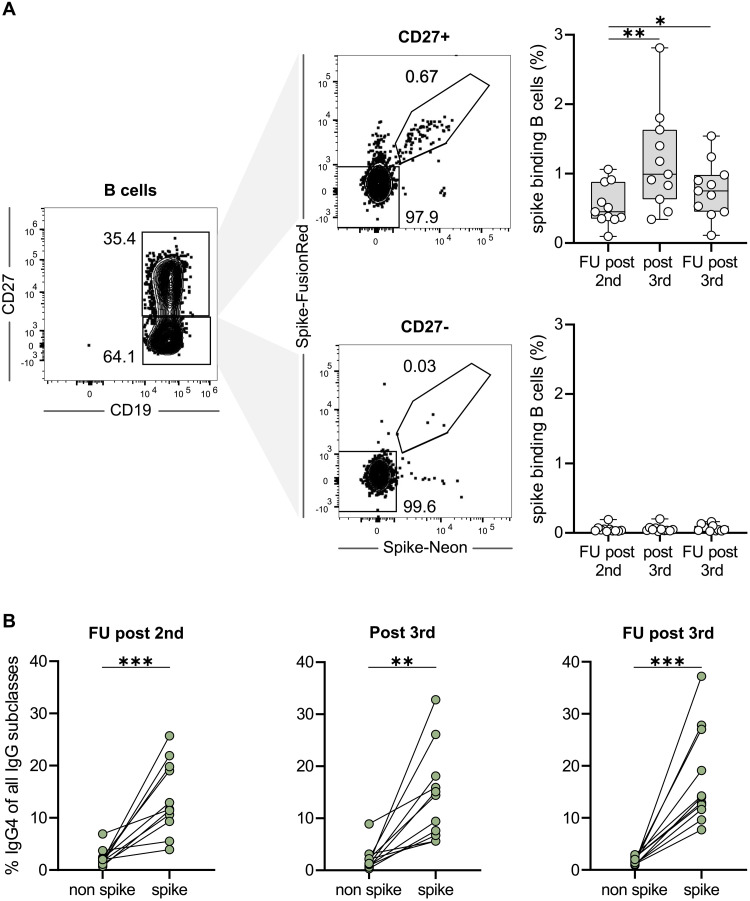
Longitudinal monitoring of class-switching in SARS-CoV-2-specific memory B cells. PBMCs from 11 volunteers of cohort 1 were analyzed for the IgG subclass contribution of spike-binding memory B cells at the indicated time points (follow-up post second, 10 days post third and follow-up post third). (**A**) Flow cytometric gating for CD19-postive and then either CD27-positive or negative B cells (left panel), binding simultaneously to recombinant Spike-Neon and Spike-FusionRed proteins (middle panel). Percentages of spike-binding cells among CD27-positive and negative B cells are summarized on the right panels. (**B**) Pairwise comparison of the contribution of IgG4 subclasses on spike-binding memory B cells versus non-binding memory B cells at three different time points. Percentages were calculated from IgG4 binding cells and the sum of cells of all 4 IgG subclasses. * p < 0.05, ** p < 0.01, *** p < 0.001; paired t-Test.

The appearance of anti-S IgG4 antibodies late after the second immunization suggests that a long period of ongoing B-cell maturation might come along with increased CSR towards distal IgG subclasses resulting in the generation of IgG4-switched memory B-cells over time. To validate the presence of these cells beyond serological testing, we characterized spike-specific memory B cells according to their IgG subclasses by flow cytometry in longitudinal PBMC samples of 11 individuals with representative, varying degrees of anti-S IgG4 antibodies late after the second immunization, shortly after third immunization as well as late after third immunization (Fig. 2). Spike-binding B cells were detected in considerable frequency almost exclusively within the CD27^+^ memory B cell population (Fig. 2A). While the frequency of IgG4-expressing memory cells among non-spike binding memory B cells was in the range of 1–8% as described before *(**28**)*, a significantly higher frequency of spike-binding memory B cells expressed IgG4 at all time points, reaching up to 37% of all IgG subclasses (Fig. 2B; Fig. S3). As expected from serological data, IgG3 was underrepresented and considerable frequencies of IgG2-positive memory B cells were found among spike-binding memory B cells (Table S2).

In addition to the flow cytometric analyses, we performed single-cell RNA sequencing (scRNA-seq) of spike-specific B cells from four selected donors 210 days after the second or 10 days after the third Comirnaty dose (Fig. S4). We used cellular indexing of transcriptomes and epitopes (CITE)-seq *(**29**)* to label spike- and receptor binding domain (RBD)-binding B cells and enriched for spike-binding IgG+ B cells by flow cytometry prior to scRNA-seq. In line with the flow cytometric data, 16% of the sorted spike-specific memory B-cells from all four donors showed sequences encoding the IgG4 subclass which were barely detectable among non-binders. Frequencies of IgG4 anti-spike memory B cells for individual donors as determined through scRNA-seq thereby mirrored serological IgG4 anti-spike levels (Fig. S4B). Of note, among all spike- and RBD-binding B cells identified, B cells with an IgG4 subclass were not phenotypically different from B cells with other IgG subclasses (Fig. S4C).

To confirm the spike-specificity of the IgG4-producing B cell clones (Fig. S4D), four recombinant monoclonal antibodies were cloned from the obtained B cell receptor (BCR) sequences, expressed in eukaryotic cells and tested for binding to RBD and full-length spike. All four monoclonal antibodies (mAbs) were able to bind to the spike protein, and one clone recognized RBD, which was in accordance with the CITE-seq results (Fig. S4E).

In summary, flow cytometry and scRNA-seq confirmed a high frequency of IgG4 spike-binding memory B cells immediately before the third immunization, which even increased thereafter.

### The IgG4 subclass does not prevail after repeated vaccination with tetanus toxoid or respiratory syncytial virus infection

Generally, IgG4 responses have been rarely observed even after repeated immunizations or infections. To corroborate this, we analyzed tetanus-specific antibody responses in 23 volunteers who had received several doses (2–16, median 6) of a tetanus toxoid (TT) vaccine (Table S3). Sera were tested for TT-specific total IgG or IgG4 antibodies using an ELISA format. TT-specific IgG4 were detectable in 9 of 23 sera, albeit at very low levels, and no correlation was found with the number of vaccinations received (Fig. S5A, B). Additionally, we tested ten individuals from our cohort 2 (Table 1) for the presence of antibodies against the respiratory syncytial virus (RSV), a respiratory pathogen that regularly causes re-infections in humans. While we found RSV-F protein-specific IgG1 antibodies in all tested sera, IgG4 was not detected (Fig. S5C). These findings support the notion that class-switching to IgG4 is not a general consequence of repeated antigen exposure in form of vaccinations or infections.

### IgG4 occurrence correlates with increased avidity, but decreased antibody effector function in an independent cohort of SARS-CoV-2 mRNA vaccinees

**Fig. 3.**
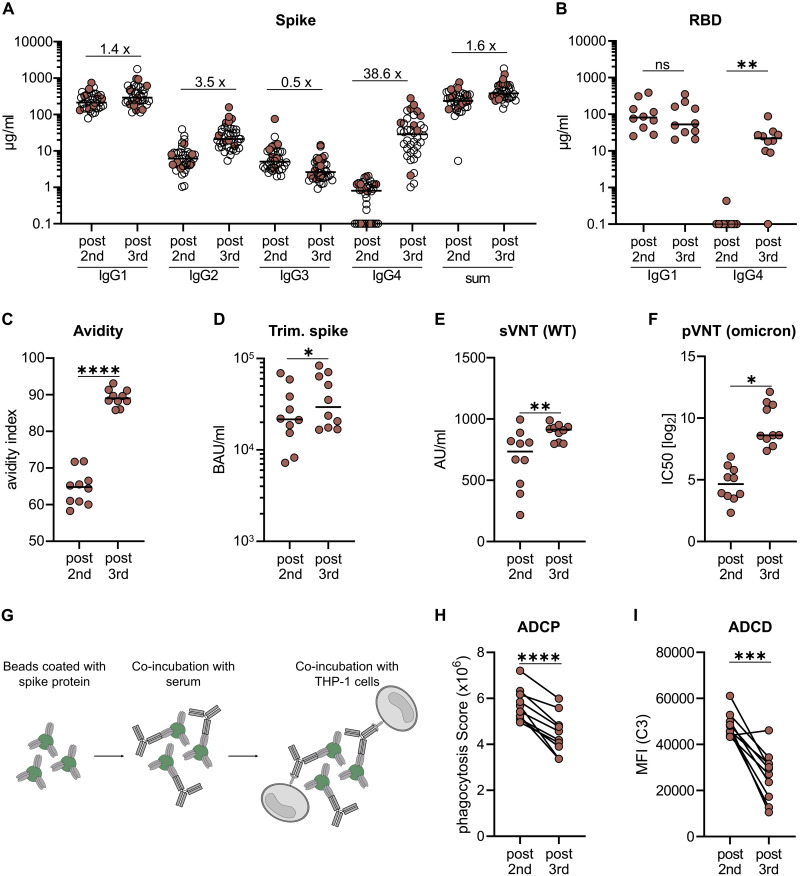
Comparison of functional antibody responses after two or three mRNA vaccinations. From a second cohort of 38 vaccinees having received three immunizations with Comirnaty (see Table 1, cohort 2), we selected ten persons to characterize the vaccine-induced antibody profile (indicated by filled circles). Only one individual in this cohort showed a positive nucleocapsid serology, despite no anamnestic clinical breakthrough infection, and was not included in the smaller sub-cohort used for functional testing. Paired serum samples collected after the second (post second) or the third (post third) vaccination were analyzed. The IgG subclass distribution measured in the flow cytometric assay and the sum of all IgG are shown for the whole cohort (**A**). The amounts of RBD-specific IgG1 and IgG4 (**B**) and the avidity (**C**) were determined by ELISA. For avidity measurements, sera were normalized according to total anti-S IgG levels and equal amounts of specific IgG were used. A fully automated CLIA assay was used to measure antibodies binding to trimeric spike protein. Antibody levels were quantified according to the WHO International Reference standard and given as BAU/ml (**D**). The neutralizing capacity was determined in a surrogate VNT against WT (**E**) and in a pseudotype VNT against the Omicron VOC (**F**). Antibody-dependent phagocytosis by the monocytic THP-1 cell line (**G**) was analyzed by using either monoclonal RBD antibodies of the different subclasses (Fig. S7) or the paired sera. The phagocytosis score is calculated as follows: % of THP-1 bead-positive x mean fluorescence intensity of bead-positive (**H**). Antibody-dependent complement deposition was analyzed on spike-coated microbeads after incubation with the paired sera. C3 deposition was detected by fluorescently labeled antibodies and mean fluorescence intensity of complement-loaded beads are shown (**I**). Circles represent individual sera and solid lines indicate the median (H, I). Statistical comparison for the two time points were done by a paired T-test. *p < 0.05, **p < 0.01, ***p < 0.001, ****p < 0.0001, and n.s. indicates not significant.

To exclude any unrecognized bias or specific characteristics of the initially described cohort 1 (Table 1, Fig. 1), a second cohort of 38 volunteers was analyzed, who had received three doses of Comirnaty using a very similar vaccination schedule (Table 1, cohort 2). Only one individual in this cohort showed a positive nucleoprotein serology, despite no anamnestic clinical breakthrough infection, and was not included in a smaller sub-cohort used for functional testing. Sera taken shortly after the second or the third vaccination (2 to 5 weeks) showed a comparable spike-specific antibody subclass distribution (Fig. 3) as described before (Fig. 1). Again, there was a substantial increase in IgG4 (38.6-fold) levels after the third immunization, whereas e.g. spike-specific IgG3 did not reach the levels seen after the second dose (Fig. 3A; Fig. S6).

Since the total amount of anti-spike IgG antibodies was only moderately (1.6-fold) elevated after the third compared to the second vaccine dose, we next investigated whether the increased proportion of IgG4 antibodies had functional consequences. To this end, paired sera from a representative subcohort of ten volunteers were analyzed. First, RBD-specific IgG1 and IgG4 antibodies were determined via ELISA. IgG4 levels were significantly increased after the third vaccination, whereas the levels of RBD-binding IgG1 were not different at the two time points (Fig. 3B). The avidity was clearly increased after the third vaccination (Fig. 3C), which is in line with recent reports *(**9**)*. Furthermore, the capacities to bind trimeric spike protein (Fig. 3D) and to prevent soluble RBD binding to ACE2 (Fig. 3E), which both serve as surrogate markers for virus neutralization, were increased after the third dose. Accordingly, this translated into superior neutralization of lentiviral (LV) particles pseudotyped with spike proteins derived from the Omicron VOC (Fig. 3F). In conclusion, repeated vaccination improved antibody effector functions mediated through the variable domain.

However, IgG2 and IgG4 are considered to have a lower potential to mediate FcγR-dependent secondary effector function *(**20**)*. Therefore, we performed an ADCP assay with the monocytic THP-1 cell line *(**30**)* (Fig. 3G). Using fluorescently labeled microbeads loaded with spike protein as targets and equal amounts of our recombinant monoclonal anti-RBD antibodies, we confirmed that IgG3 and IgG1 are more potent in inducing phagocytosis than IgG4 and IgG2 (Fig. S7). Using FcγRIIA, FcγRIIB or FcγRIII-expressing reporter cells *(**31**)*, engagement of IgG2 and IgG4 results in reduced activation of the FcγRIIA, which was reported to be a key mediator of ADCP *(**30*, *32**)* (Fig. S7). Consistent with this, sera taken after the third vaccination and normalized to the amount of anti-spike antibodies yielded significant lower phagocytic scores than sera from the same donors after two immunizations (Fig. 3H). Furthermore, antibody-dependent complement deposition on spike-coated microbeads was also significantly reduced after incubation with sera taken after the third vaccination (Fig. 3I). Together, these data show that spike protein-reactive IgG2 and IgG4 exhibit reduced Fc-mediated effector functions.

### Impact of breakthrough infections on vaccine-induced antibody responses

The fact that individuals, who experienced a breakthrough infection after being three times vaccinated with mRNA, showed the highest IgG4 levels in cohort 1 (Fig. 1) suggested that infections with SARS-CoV-2 can also activate IgG4-switched memory B cells. To investigate this in more detail, we identified twelve persons from a study cohort of breakthrough infections (CoVaKo study), who were vaccinated two or three times with SARS-CoV-2 mRNA vaccines and who experienced a breakthrough infection 25 to 257 days after the second or 57 to 164 days after the third mRNA vaccination (Table S4). Serum samples were collected on the day of study inclusion (visit (V) 1, typically within the first week) as well as two (V2) and four weeks (V4) after infection-confirming PCR.

In all individuals, we detected an anamnestic antibody response with an increase in spike-binding antibodies from V1 to V4 irrespective of IgG subclasses (Fig. S8). Consistent with our previous findings, IgG4 levels were generally higher in individuals having received three compared to two mRNA vaccinations. Interestingly, in the cohort that had two mRNA vaccinations before breakthrough infections, only three individuals developed IgG4 antibodies that were above the lower limit of quantification. These three individuals experienced the infection with the largest time difference to the last vaccination, at 95, 201 or 257 days after the second vaccination, while in the other nine patients the infection took place between 25 and 78 days after the second mRNA shot. This supports the hypothesis that the switch to IgG4 is a consequence of ongoing GC maturation and that it takes several months until IgG4-switched memory B cells appear.

## DISCUSSION

In the present study we longitudinally tracked the antibody response in volunteers vaccinated with two or three doses of Comirnaty for a period of at least 8 months after the first vaccination. We found an mRNA vaccine-driven expansion of memory B cells expressing IgG4. We detected spike-specific IgG4 antibodies in about half of the serum samples collected five to seven months after the second immunization, all of which did not show any IgG4 at earlier time points. For all other IgG subclasses, a decline was seen in the same period. Moreover, after the third immunization, IgG4 levels sharply increased and became detectable in almost all vaccinees.

In line with the proposed ongoing GC reaction, the appearance of IgG4 antibodies might be a consequence of consecutive events of CSR and the maturation of IgG4-switched memory B cells. IgG3 antibodies were less efficiently boosted and did not reach the levels seen after the second dose. Considering the order of the four γ heavy chain genes (γ3-γ1-γ2-γ4) within the immunoglobulin gene complex on chromosome 14 *(**21**)*, this would support the hypothesis of consecutive CSR from proximal IgG3 to distal IgG4 *(**33*, *34**)*. Interestingly, it is reported for the adult immune repertoire that CSR towards IgG2 or IgG4 is more frequently occurring from IgG1 B cells than from IgM/IgD cells *(**34**)*.

When we isolated spike-specific memory B cells from vaccinees 210 days after the second vaccination as well as ten days and 5 months after the third vaccination, we confirmed by flow cytometry and single-cell sequencing the presence of substantial numbers of spike-reactive, IgG4 switched B cells, whereas IgG3-positive clones were hardly detectable. We cannot formally rule out *de novo* class-switching towards IgG4 immediately after booster vaccination. However, the presence of IgG4 antibodies in the sera at that time point, together with the rapid rise of IgG anti-spike serum antibodies, supports the idea of a reactivation of already present IgG4 memory B cells through the booster immunization.

In a cohort of breakthrough infections, the anamnestic IgG4 antibody response correlated with the time interval between immunization and infection. Individuals having experienced a breakthrough infection within the first 70 days after the second vaccination did not have substantial serum levels of anti-spike IgG4 at their first visit, which also did not significantly increase during the following observation period. In contrast, anamnestic IgG4 responses were seen when breakthrough infections occurred later than 3 months after the second immunization, and were robustly detectable when the study participants had been vaccinated three times before infection. Although the number of subjects studied was limited and we did not stratify for potential confounding factors (e.g., the initial viral load, severity of disease or the VOC causing the infection), the presented data are consistent with the hypothesis of a slowly developing pool of IgG4-switched memory B cells after two doses of mRNA vaccines. Furthermore, we observed significantly higher IgG4 levels after two doses of Comirnaty mRNA vaccine compared to a heterologous immunization regimen with a primary Vaxzevria vaccination followed by one dose of Comirnaty, although the total anti-spike IgG response was comparable. This argues against the hypothesis that repeated exposure to the spike protein itself triggers the unusual IgG4 response. It is currently not clear whether or to what extent the Comirnaty mRNA vaccination or the short interval of immunizations are responsible for the observed long-lasting GC reactions *(**12*–*14**)*, but a prolonged presence of vaccine mRNA or antigen in the lymph node might be a potential explanation *(**12**)*. Furthermore, a robust and persistent T follicular helper cell (T_FH_) response for up to six months after mRNA vaccination has been described in draining lymph nodes *(**17**)*, which might be involved in the regulation of CSR by recurrent interactions of GC B cells with T_FH_ cells. Of note, our study was restricted to vaccinees receiving the Comirnaty vaccine. Since the quantities and the functional profile of spike-specific antibodies induced by Comirnaty and mRNA-1273 have been reported to be slightly different *(**6*, *35**)*, it will be interesting to analyze whether repeated vaccination with mRNA-1273 induces a similar switch to non-inflammatory IgG subclasses.

Independent of the underlying mechanism, the induction of antiviral IgG4 antibodies is a phenomenon infrequently described and raises important questions about its functional consequences. Neutralizing antibodies preventing the initial binding of the viral particle to its specific cellular receptor are considered to be the most protective measure against SARS-CoV2 infections *(**36**)*. This competitive binding is mediated by the variable antigen-binding site and does not rely on the constant part of the Fc fragment. Indeed, in the present study we confirmed previous reports on improved avidity and neutralizing potential of vaccine-induced antibodies after the third vaccination *(**9*–*11**)*. However, the large number of breakthrough infections caused by the Omicron variant indicates that current vaccination regimens do not confer sterilizing protection. Once infection is established, Fc-mediated effector functions become more relevant to clear viral infections. Systemic serology approaches have even revealed that different antibody functions can contribute to various degrees to protection dependent on the viral pathogen, as shown for influenza viruses, RSV or SARS-CoV-2 *(**37*–*40**)*. Passive immunization studies in animal models have further demonstrated that the degree of protection achieved by the application of monoclonal antibodies depends on their IgG subclass *(**41*–*44**)*. In this regard, IgG4 is considered as an anti-inflammatory IgG with low potential to mediate Fc-dependent effector function such as ADCC or ADCP *(**20*, *45**)*.

High levels of antigen-specific IgG4 have been reported to correlate with successful allergen-specific immunotherapy by blocking IgE-mediated effects *(**46**)*. In addition, increasing levels of bee venom-specific IgG4 have been detected in beekeepers over several beekeeping seasons and finally even became the dominant IgG subclass for the specific antigen, i.e. phospholipase A (PLA). Interestingly, the IgG4 response is characterized by a very slow kinetics and takes several months to appear, whereas PLA-specific IgG1 antibodies were already measurable at earlier time points, which resembles our findings in this study *(**47**)*. Furthermore, an increase in PLA-specific IgG4-switched B cells was observed in patients undergoing specific immunotherapies (SIT) *(**48**)*.

So far, only few studies on the role of vaccine-induced IgG4 responses against infectious diseases are available. In the field of HIV vaccine development, repeated protein immunization in the trial VAX003 *(**49**)* led to higher levels of HIV gp120-specific IgG2 and IgG4, whereas a prime-boost immunization with a canarypox vector (ALVAC) and the same protein vaccine in the RV144 trial *(**50**)* resulted in higher HIV-specific IgG3 responses correlating with partial protection against HIV *(**51*, *52**)*. Furthermore, the vaccine-elicited IgG3 antibodies enhanced effector functions as ADCC and ADCP, but vaccine-induced IgG4 inhibited those functions *(**52**)*.

With respect to the control of viral infections, little is known regarding virus-specific IgG4 antibody responses. As shown here for RSV-specific IgG responses, IgG4 is hardly induced by acute respiratory viral infections even after repeated exposure. Although measles-specific IgG4 antibodies can be induced by natural infection *(**53**)*, even chronic viral infections like HCMV do not trigger significant specific IgG4 antibodies *(**53**)*.

There are very few reports on the induction of IgG4 after natural infection with SARS-CoV-2. The dominant subclasses were mostly IgG1 and IgG3 *(**54*–*56**)*. Nevertheless, a Brazilian study during the early phase of the pandemic correlated an early onset and high levels of anti-spike IgG4 antibodies with a more severe COVID-19 progression after SARS-CoV-2 infection, which might indicate a less effective antibody response *(**56**)*. Additionally, Della-Torre et al. reported on a significant association of high IgG4/IgG1 ratios with poor disease outcome *(**57**)*. However, in the case of a primary immune response, the causality is difficult to address, since it is also possible that a more severe infection leads to an IgG4 response and not vice versa.

In our study, antibody-mediated phagocytic activity and complement deposition were reduced in sera after the third immunization, in parallel to higher proportions of anti-spike IgG4 antibodies. However, how these changes affect subsequent virus infections remains unclear. Since Fc-mediated effector function could be critical for viral clearance, an increase in IgG4 subclasses might result in longer viral persistence in case of infection. However, it is also conceivable that non-inflammatory Fc-mediated effector functions reduce immunopathology while virus is still being neutralized via high-avidity antibody variable regions. In a cohort of vaccinees with breakthrough infections, we did not obtain any evidence for an alteration of disease severity, which was mild in almost all of our cases. Larger cohorts with differential disease severities will be needed to address this aspect in the future. However, our results clearly demonstrate that a subsequent infection can further boost IgG4 antibody levels, with IgG4 becoming the most dominant among all anti-spike IgG subclasses in some individuals.

In summary, our study demonstrates an mRNA vaccine-induced antiviral IgG4 antibody response appearing late after secondary immunization. Further investigations are needed to clarify the precise immunological mechanisms driving this response and to evaluate whether an IgG4-driven antibody response affects subsequent viral infections and booster vaccinations. This is not only relevant for potential future vaccine campaigns against SARS-CoV-2, but also for new mRNA-based vaccine developments against other pathogens.

## MATERIALS AND METHODS

### Study cohorts

The cohorts are described in detail in Table 1 and Tables S1, S3 and S4. Ethics approval was granted by the local ethics committee in Erlangen (Az. 340_21B, Az. 46_21B, 350_20B and 235-18B). All donors were of European Caucasian ethnicity.

### FACS-based antibody assay

The FACS-based antibody assay was adapted from a previously published assay to detect IgG, IgA and IgM antibodies *(**25**)*. Here, stably transduced HEK293T cells with doxycycline-dependent expression of SARS-CoV-2 spike protein derived from the Wuhan strain were used as target cells. In order to be able to quantify spike-specific IgG1, IgG2, IgG3 and IgG4, we cloned and generated unique recombinant, monoclonal antibodies for each subclass with an identical RBD-recognizing variable region. The sequence for the BCR was obtained from spike-binding B cells isolated from an infected individual during the early phase of the pandemic. The recombinant mAbs were produced in HEK 293F cells. Purified mAbs were used to generate standard curves in each assay to allow quantification of absolute levels of the respective subclass in the tested sera. For a binding assay, spike protein expression was induced by doxycycline treatment for 48 h, before 1x10^5^ cells were incubated with serum samples at various dilutions in 100 μl FACS-PBS (PBS with 0.5% BSA and 1 mM sodium azide) for 20 minutes at 4°C to bind to spike protein on the surface. After washing, bound S-specific antibodies of the different IgG subclasses were detected using the following antibodies: mouse anti-hIgG1-FITC (Sigma Aldrich,Cat# F0767, RRID:AB_259409), mouse anti-hIgG2-PE (SouthernBiotech Cat# 9070–09, RRID:AB_2796639), rabbit anti-hIgG3 (Thermo Fisher Scientific Cat# SA5–10204, RRID:AB_2665317) followed by anti-rabbit-IgG-AF647 (Thermo Fisher Scientific Cat# A-21443, RRID:AB_2535861) and mouse anti-hIgG4-Biotin (SouthernBiotech Cat# 9190–08, RRID:AB_2796685) followed by Streptavidin-PB (ThermoFisher, S11222). After further washing, samples were measured on an Attune NxT flow cytometer (ThermoFisher) and analyzed using FlowJo software (Tree Star Inc.). The median fluorescence intensities (MFI) correlate with the level of bound antibodies and standard curves for the different subclasses were generated by the statistical analysis software Graph Pad Prism 9 (GraphPad Software, USA) using 4-Pl plotting. The median MFI of three negative sera were used to set the background for each subclass. To visualize all sera in the graphs, we set all sera with MFI values below the background to 0.1 μg/ml. The lowest limit of quantification is indicated in each graph and represents the lowest amount of the respective standard mAbs that were detected. (1.56 μg/ml for IgG2, IgG3 and IgG4; 5.6 μg/ml for IgG1 for a 1:100 dilution of the sera).

For the detection of RSV-specific antibodies, stably transduced HEK 293 T cells with doxycycline-inducible expression of RSV F-protein applying the same protocol as described above. The only exception was that there were no monoclonal standard Abs available. Thus, the MFI were plotted in the figure with different background values as stated in the figure legend.

### Automated measurement of trimeric spike antibodies

Sera collected from patients with breakthrough infections were analyzed for anti-spike antibodies using the fully automated LIAISONSARS-CoV-2 TrimericS IgG assay (DiaSorin, Saluggia, Italy) according to the manufacturer’s instructions. Antibody levels were quantified using the WHO International Reference standard and given as BAU/ml. An antibody level above 33.8 BAU/ml was considered as positive.

### Surrogate virus neutralization assay

For the detection of neutralizing antibodies, we ran the iFlash-2019-nCoV NAb assay on the iFlash-1800 CLIA Analyzer (YHLO Shenzhen, China) according to the manufacturer’s instructions. It detects antibodies that are able to compete with RBD binding to the SARS-CoV-2 receptor ACE2. According to the WHO standard, the neutralizing activity is given in AU/ml and the linear range for positive results is between 10 and 800 AU/ml.

### Antigen-specific antibody ELISA and avidity measurement

RBD-specific antibodies of the subclasses IgG1 and IgG4 were analyzed by ELISA. To this end, ELISA plates were coated with 100 ng of the RBD peptide (provided by Diarect GmbH, Freiburg) in 100 μl carbonate buffer (50 mM carbonate/bicarbonate, pH 9.6) per well over night at 4°C. Free binding sites were blocked with 5% skim milk in PBS-T (PBS containing 0.05% Tween-20) for 1 h at RT. Serum samples were diluted 1:100 or 1:1,000 in 2% skimmed milk in PBS-T and incubated on the plate for one hour at RT. Recombinant monoclonal RBD antibodies were used as standard to allow the absolute quantification. After three washing steps with 200 μl PBS-T, anti-hIgG1-Biotin (Thermo Fisher Scientific Cat# MH1515, RRID:AB_2539710, dilution 1:2,000) or anti-hIgG4-Biotin (SouthernBiotech Cat# 9190–08, RRID:AB_2796685, dilution 1:5,000) were added for 1 h at RT followed by an incubation with HRP-coupled streptavidin (1:2,000, ABIN376335, antibodies-online.com). Subsequently, the plates were washed seven times with PBS-T and after the addition of ECL solution, the signal was measured on a microplate luminometer (VICTOR X5, PerkinElmer) and analyzed using PerkinElmer 2030 Manager software.

To estimate the avidity of the anti-RBD antibodies, sera were normalized for their anti-sike antibody content and 100 ng of anti-spike antibodies were used in the RBD avidity ELISA. This time, antigen-antibody complexes were incubated in the presence of 1.0 M ammonium thiocyanate or PBS as control for 30 min at room temperature. After washing to remove antibodies bound with low avidity, the ELISA was completed as described before. HRP-coupled anti-hIgG were used for the detection of bound antibodies. The relative avidity index was calculated as [IgG concentrations (NH_4_SCN) / IgG concentrations (PBS)] x 100, and is given in percent.

For the estimation of tetanus toxoid-specific antibodies, the same protocol as for the RBD ELISA was applied, but 0.5 μl/well of the inactivated TT vaccine (Tetanol Pur, Novartis) was used for coating. For the detection of TT-specific antibodies, either HRP-coupled anti-hIgG or anti-hIgG4-Biotin (SouthernBiotech Cat# 9190–08, RRID:AB_2796685, dilution 1:5,000) were used followed by incubation with HRP-coupled streptavidin.

### Pseudotype neutralization assay

Neutralization of recently emerged Omicron B1 variant was assessed with the help of spike-pseudotyped simian immunodeficiency virus particles as described before *(**58**)*. To produce pseudotyped reporter particles, HEK293T cells (RRID:CVCL_0063) were transfected with the SIV-based self-inactivating vector encoding luciferase (pGAE-LucW), the SIV-based packaging plasmid (pAdSIV3) and the Omicron spike-encoding plasmid.

For the assessment of pseudotype neutralization, HEK293T-ACE2 cells were seeded at 2x10^4^ cells/well in a 96-well flat bottom plate. 24 h later, 60 μl of serial dilutions of the serum samples were incubated with 60 μl lentiviral particles for 1 h at 37°C. HEK293T cells were washed with PBS and the particle-sample mix was added to the cells. 48 h later, medium was discarded, and the cells washed twice with 200 μl PBS. Following 50 μl PBS and 25 μl ONE-Glo (Promega Corp, Madison, USA) was added and after 3 minutes the luciferase signal was assessed on a microplate luminometer (VICTOR X5, PerkinElmer) and analyzed using PerkinElmer 2030 Manager software. The reciprocal serum ID50 was determined with Prism GraphPad 9 (San Diego, California, USA) by application of the Sigmoidal 4PL function.

### FcγR activation reporter assay

The assay used for testing IgG-dependent activation of FcγRs is based on BW5147 (RRID:CVCL_3896) reporter cells stably expressing chimeric FcγR-ζ chain receptors which stimulate mouse IL-2 production in the presence of Ag-IgG immune complexes, provided that the opsonizing IgG is able to crosslink the particular FcγR *(**31**)*. First, ELISA plates pre-coated with spike protein (kindly provided by InVivo BioTech Services GmbH, Hennigsdorf, Germany) were used to quantify the binding capacity of the different RBD-specific monoclonal antibodies. Ten-fold serial dilutions of the different mAbs (10 μg/ml to 0.001 μg/ml) were added to the plates and incubated 1 h at 37°C. After washing, plates were incubated with biotinylated anti-human IgG (1 h, 37°C) followed by washing, addition of Streptavidin HRP (30 minutes at RT) and TMB substrate. Binding was quantified by measuring OD values at 450 nm and by calculating the area under the curve (AUC ELISA) To determine FcγR activation, serially diluted mAbs were incubated for 30 minutes at 37°C on spike pre-coated plates. Plates were thoroughly washed with RPMI 10% (v/v) to remove non-immune IgG and individual BW:FcγR-ζ reporter cells (expressing human CD16A, CD32A, CD32B, CD64), were added to the spike-IgG complexes formed in the ELISA plate and incubated for 16 h at 37°C, 5% CO_2_. Afterwards, mouse IL-2 secretion was measured by anti-IL-2 ELISA, using purified rat anti-mouse IL-2 (BD-Pharmingen,) and biotin rat anti-mouse IL-2 (BD-Pharmingen, 1:500) *(**31**)*. OD values were measured at 450 nm and the area under the curve was calculated (AUC IL-2). The IL-2 production in the supernatants was quantified as previously described*(**31**)*. The level of IL-2 as measure of the respective FcγR activation was first normalized to the total amount of spike binding antibodies, e.g. AUC IgG1 IL-2_CD16_/AUC IgG1 ELISA. Furthermore, the activation of the different FcγR by the IgG2, IgG3 and IgG4 subclasses were normalized to the respective activation by the IgG1 mAb to allow for inter-assay comparisons.

### Antibody-dependent phagocytosis and complement deposition

The phagocytosis assay was adapted from Ackerman et al. *(**30**)*. Yellow-green fluorescent beads (FluoSpheres NeutrAvidin-labeled Microspheres, 1.0 μm; ThermoFisher, cat# F8776) were coated with biotinylated SARS-CoV-2 S1 spike protein (GenScript, cat# Z03501) at a ratio of 100 ng protein per 5x10^8^ beads overnight at 4°C in FACS-buffer (PBS containing 0.5% bovine serum albumin and 1 nM sodium azide). After washing the beads with FACS-buffer, 5x10^6^ beads were seeded in 10 μl FACS-buffer per well in a 96-well plate. Monoclonal, spike-specific antibodies (1 ng mAb /well) or serum dilutions normalized to their spike-specific IgG concentration (1 ng anti-S /well) were added in a volume of 10 μl FACS-buffer. Sera were heat-inactivated at 56°C for 30 min. After an incubation of two hours at 37°C, 10^5^ THP-1 cells (ATTC TIB-202, RRID:CVCL_0006) were added to the beads in a volume of 100 μl RPMI 1640 supplemented with 10% FCS, 2 mM L-Glutamine, and 1% penicillin/streptomycin and the plates were incubated for 16 hours at 37°C. Cell-bead mixtures were washed two times with 180 μl PBS (400xg, 3 min) before treatment with 180 μl 0.25% Trypsin/0.02% EDTA for 10 minutes. After two additional washing steps with FACS-buffer, samples were resuspended in 200 μl FACS buffer and subjected to flow cytometry. Phagocytosis was assessed by first gating on THP-1 cells and then selecting cells that show yellow-green fluorescence of the phagocytosed beads. The phagocytosis score was calculated as follows: % of bead-positive THP-1cells × mean fluorescence intensity of bead-positive.

The complement deposition assay was adapted from *(**59**)*. Spike-coated beads were prepared as described for the phagocytosis assay. 5x10^6^ beads were then incubated for two hours at 37°C with serum samples normalized to their spike-specific antibody concentration (100 ng IgG/well) in a total volume of 20 μl FACS buffer in a U-bottom 96 well plate. After two washing steps with FACS buffer, beads were incubated for 15 minutes at 37°C with Guinea pig complement (Cedarlane Lab, cat# CL4051) diluted 1:50 in 200 μl RPMI supplemented with 10% FCS per well. Following two more washing steps, goat polyclonal anti-guinea pig complement C3 (MP Bio Cat# 0855371, RRID: AB_2334449, 1:100 in 50 μl FACS-buffer) was added to the beads for 15 minutes at RT. Subsequently, beads were washed twice before adding donkey anti-goat IgG H&L-AF647 (Abcam Cat# ab150135, RRID:AB_2687955) for 15 minutes at RT. Complement deposition was finally assessed after two more washing steps by measuring the AF647 fluorescence intensity of the beads. Data were acquired on an Attune NxT flow cytometer (ThermoFisher) and analyzed using FlowJo software (Tree Star Inc.).

### Isolation and cryopreservation of peripheral blood mononuclear cells (PBMC)

PBMCs were isolated from citrated peripheral blood of vaccinated individuals by density gradient centrifugation using BioColl separating solution, density 1.077 g/ml (Bio&SELL) and frozen in heat-inactivated FCS + 10% DMSO (Sigma-Aldrich) for liquid nitrogen storage.

### Flow cytometric detection of spike-protein binding B cells

For labelling of spike-specific memory B cells in flow cytometry a simultaneous staining with two trimeric, prefusion stabilized spike proteins *(**60**)* fused to NeonGreen or FusionRed was used. The spike ectodomain sequence was cloned into a pMT vector encoding a BiP signal peptide for efficient translocation, a FusionRed gene for flow cytometric detection of spike-specific B cells and a C-terminal double Strep tag for efficient affinity purification of spike trimers. Protein expression was carried out in *Drosophila melanogaster* Schneider 2 cells as described before *(**61**)*. Trimeric Spike protein was purified by affinity chromatography from the supernatant using a Strep-Tactin Superflow column (IBA, Goettingen, Germany) followed by gel filtration chromatography using a Superose 6 Increase 10/300 column (Cytiva). B cells were stained with antibodies for CD19-BV421 (BioLegend Cat# 302233, RRID:AB_10897802), and CD27-PE-Cy7 (BioLegend Cat# 356412, RRID:AB_2562258). For the detection of IgG subclasses, the following antibodies were applied in 2 separate panels using BV650 conjugated anti-hIgG1 (clone 6001, BD Biosciences) and rabbit anti-hIgG3 (Thermo Fisher Scientific Cat# SA5–10204, RRID:AB_2665317) followed by anti-rabbit-IgG-AF647 (Thermo Fisher Scientific Cat# A-21443, RRID:AB_2535861) in both panels and either anti-hIgG4-Biotin (SouthernBiotech Cat# 9190–08, RRID:AB_2796685) or anti-hIgG2-biotin (BD Biosciences Cat# 555874, RRID:AB_396190) followed by BV510-conjugated streptavidin (BioLegend, Cat# 405234).

### Single B cell sequencing analysis

For labelling of spike-specific memory B cells a trimeric, prefusion stabilized spike protein *(**60**)* fused to FusionRed was used.

For CITE-seq a unique molecular 5′ feature barcode was added to purified spike-FusionRed protein by the 5′ feature Barcode Antibody conjugation Kit – Lightning Link (Abcam) exactly as described by the manufacturer. RBD was also labelled by a different feature barcode.

Live CD19^+^ IgG^+^ spike-FusionRed-binding memory B cells were sorted on a MoFlo Astrios Cell Sorter in the FACS-Core facility of the FAU. FusionRed-negative CD19^+^ IgG^+^ B cells were sorted in parallel and mixed with spike sorted B cells. FACS staining solution also contained barcoded hashtags for sample identifications (BioLegend) as well as barcoded RBD. Libraries for single-cell transcriptome sequencing and scBCR-sequencing were prepared using the Chromium Single-Cell 5′ Library Gel Bead and Construction Kit, v2 Dual index Chemistry Kit and Chromium Single-Cell V(D)J BCR Enrichment Kit (10x Genomics, CA, USA). The scRNA-seq, CITE-seq and VDJ libraries were sequenced on the Illumina HiSeq-2500 platform in the NGS-Core facility of FAU. The scRNA-seq reads were aligned to the human reference genome GRCh38 (UCSC, CA, USA), after generation of cell-gene matrices via Cell Ranger v6.1.2 (10x Genomics). Cell Ranger v6.1.2 multi pipeline was applied for scRNA-seq and VDJ-seq analysis, while CITE-seq-Count v1.4.5 was applied to count the surface features*(**29**)*. Data analysis was further pursued with the R package Seurat v4.1.1*(**62**)* under R v4.2.0. Cells with high mitochondrial gene content (>5%) were filtered out. Doublets and unlabeled samples were identified and filtered utilizing sample hashtags. For further analysis of antibody VDJ genes the IMGT/V-QUEST platform was used*(**63**)*.

### Expression of recombinant antibodies

From the V_H_ and V_L_ sequences obtained by scRNA analysis, synthetic gBlocks were synthesized (IDT) and cloned into expression vectors for human IgG1 and human Igκ, essentially as described by Tiller et al.*(**64**)*, except that we used Gibson assembly for the cloning. Heavy and light chain plasmids were transfected into 293 cells and antibodies were harvested from the supernatants on day 3 after transfection.

## Supplementary Materials

### Other Supplementary Material for this manuscript includes the following:

Data file S1

MDAR Reproducibility Checklist

## Notes

View/request a protocol for this paper from *Bio-protocol*.
