# Supplementary material for: Class switch towards non-inflammatory, spike-specific IgG4 antibodies after repeated SARS-CoV-2 mRNA vaccination
Source: Sci Immunol. 2022 Dec 22:eade2798. doi: 10.1126/sciimmunol.ade2798 (PMC9847566; doi:10.1126/sciimmunol.ade2798)
Supplement: Supplementary file 1 — Figs. S1 to S8 Tables S1 to S4 [file sciimmunol.ade2798_sm.pdf]

Science Immunology
AAAS

Supplementary Materials for

Class switch towards non-inflammatory, spike-specific IgG4 antibodies after repeated SARS-CoV-2 mRNA vaccination

Pascal Irrgang *et al.*

Corresponding author: Kilian Schober, kilian.schober@uk-erlangen.de; Thomas H. Winkler, thomas.winkler@fau.de; Matthias Tenbusch, matthias.tenbusch@fau.de

Sci. Immunol. **7**, eade2798 (2022)
DOI: 10.1126/sciimmunol.ade2798

The PDF file includes:

Figs. S1 to S8
Tables S1 to S4

Other Supplementary Material for this manuscript includes the following:

Data file S1
MDAR Reproducibility Checklist

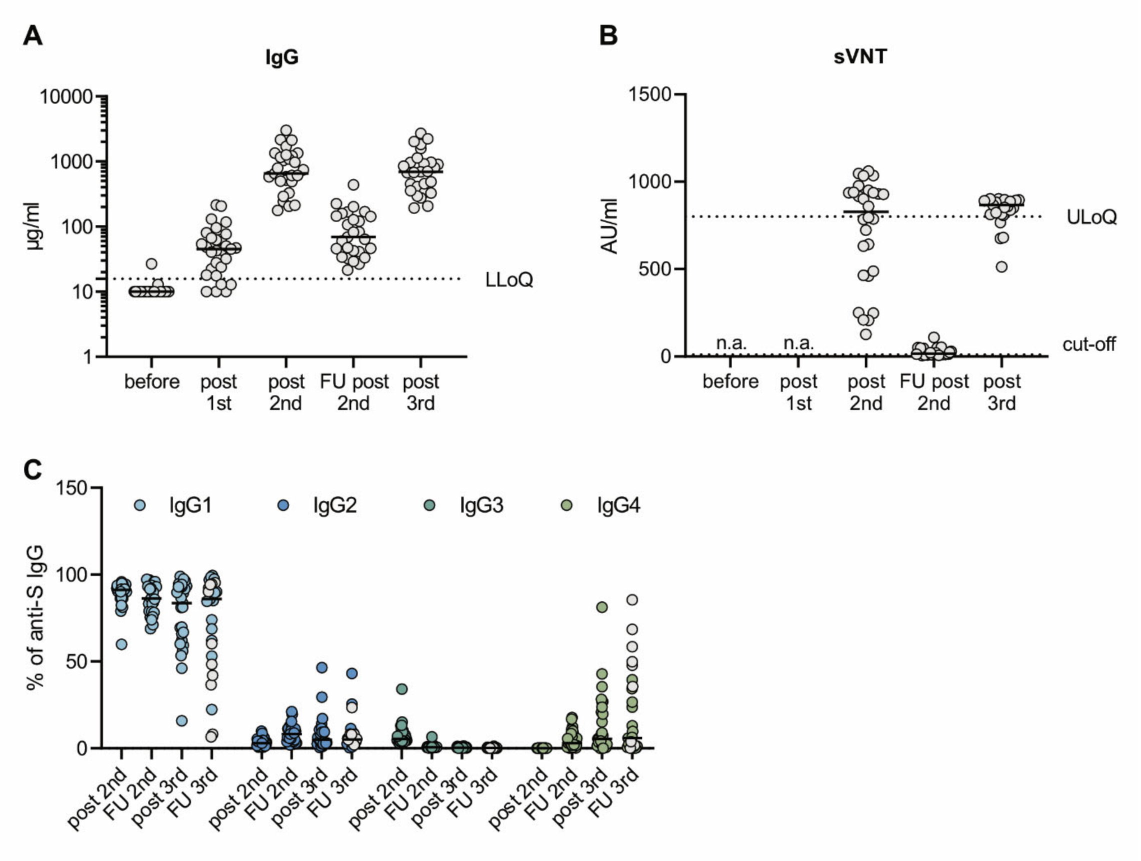

Figure S1: Longitudinal analyses of vaccine-induced antibody responses

29 volunteers received three doses of the mRNA vaccine Comirnaty as detailed in Table 1. Serum samples were collected at a median of ten days after each vaccination (post 1st, post 2nd, post 3rd) and during a follow-up visit at 210 days after the second vaccination (FU 2nd). (**A**) The total amount of spike-specific IgG was quantified by a flow cytometric assay using cell lines expressing full-length spike protein as targets. (**B**) The neutralizing capacity was measured in a fully-automated surrogate virus neutralization assay. The latter was considered as positive within a linear range from 10-800 AU/ml. The dotted line indicates the cut-off value. (**C**) The relative abundance of the different anti-S IgG subclasses is shown for each individual for the indicated time points. Individuals who have experienced a breakthrough infection between the two time points post 3rd and FU 3rd are indicated by grey dots. Dots represent individual donors with the respective median.

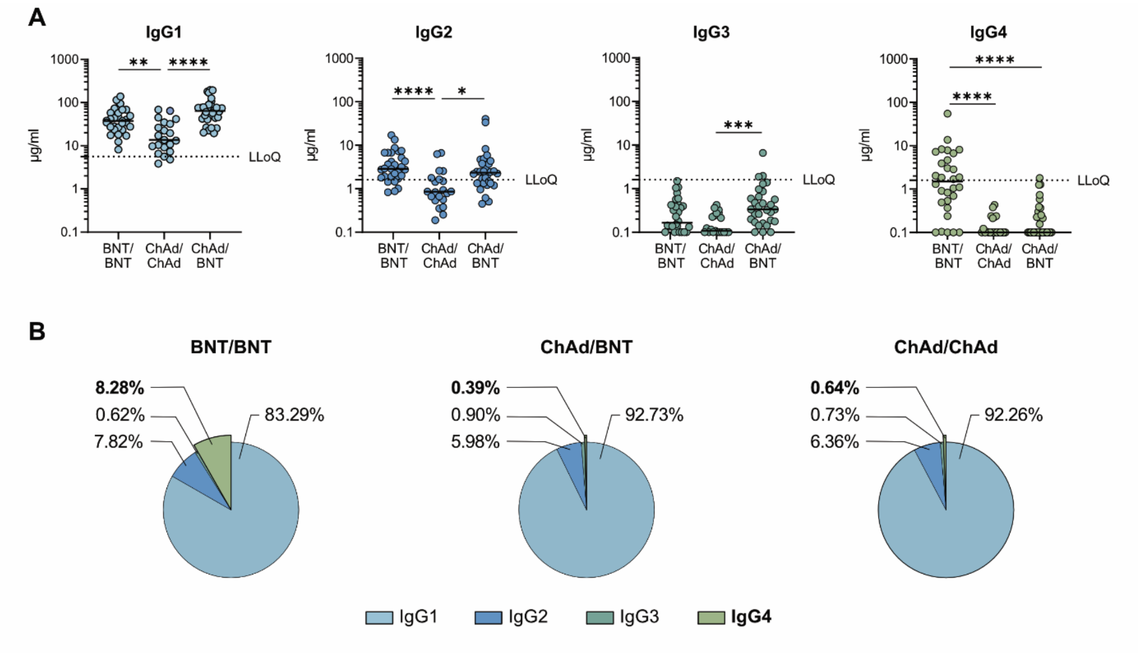

Figure S2: Comparison of IgG subclass specific antibody responses

Spike-specific antibody responses were analyzed in 81 individuals around five months after the second vaccination (Supplementary Table 1). 21 individuals had received two doses of the adenoviral vector vaccine (Vaxzevria) 30 had received an initial prime immunization with Vaxzevria followed by a boost with Comirnaty and an additional 30 were vaccinated twice with Comirnaty. (**A**) The different IgG subtypes were quantified by flow cytometry. For visualization purposes, sera with MFI values below the background were set to 0.1 µg/ml. The lowest limit of quantification (LLoQ) is indicated in each graph by a dotted line and represents the lowest detectable amount of the respective standard mAbs (1.56 µg/ml for IgG2, IgG3 and IgG4; 5.6 µg/ml for IgG1). All individual values are depicted by open circles and the median is presented by the line. Kruskal-Wallis followed by Dunn's multiple comparisons test was used for inter group statistics. *$p < 0.05$, **$p < 0.01$, ***$p < 0.001$, ****$p < 0.0001$. (**B**) The proportion of the different IgG subclasses of the total anti-S IgG response is shown for the different vaccination regimens. Depicted are the means of each IgG subclass.

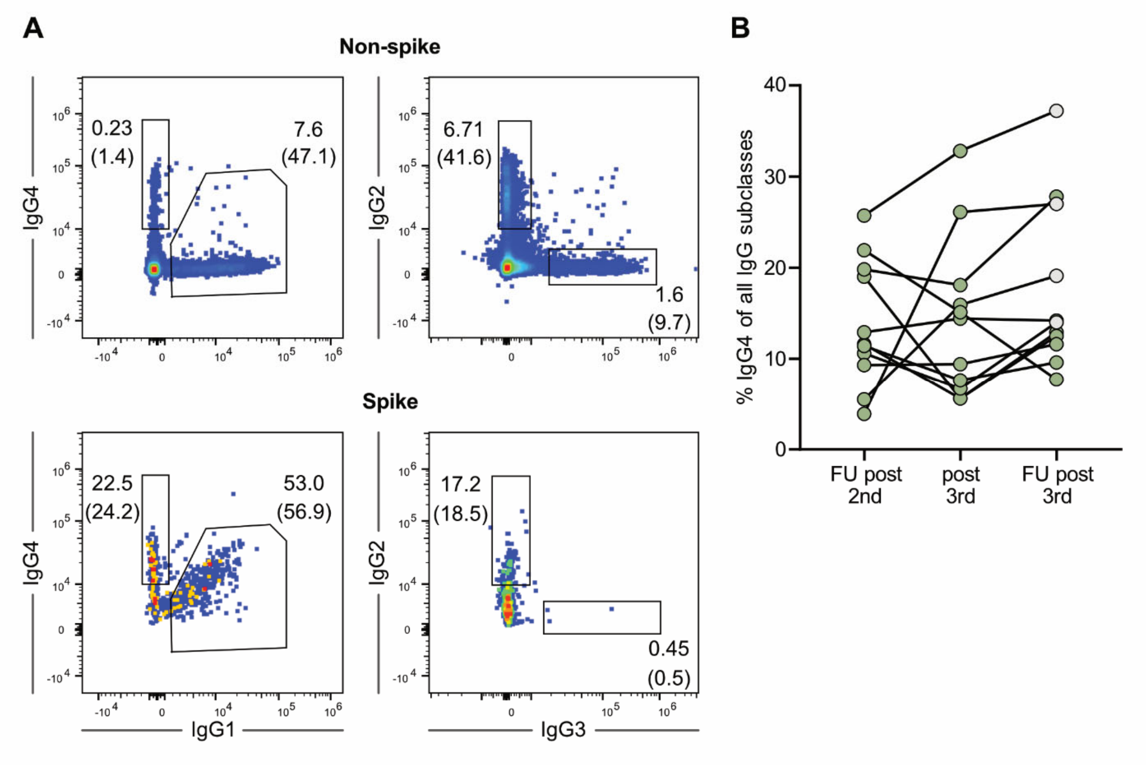

Figure S3: IgG subclass expression of SARS-CoV-2-specific memory B cells

(**A**) Flow cytometric analysis of $CD19^{+}CD27^{+}$ memory B cells. Representative dot plots of memory B cells which do not bind (upper panel) or bind (lower panel) the labelled spike protein (see Fig. 2) are shown for an individual donor. Surface expression of different IgG subclasses (IgG1 vs. IgG4 and IgG2 vs. IgG3) was determined and percentages of positive cells are indicated next to the gates. Since IgA^{+} and IgM^{+} B-cells are also present in the $CD19^{+}CD27^{+}$ memory compartment, the percentages of the respective IgG subclass among the sum of all IgG subclasses are also indicated in parentheses. (**B**) Longitudinal analysis of IgG4 subclass contribution of spike-binding memory B cells. Individuals that had experienced a breakthrough infection in the time frame between post 3rd and FU 3rd are indicated by grey circles.

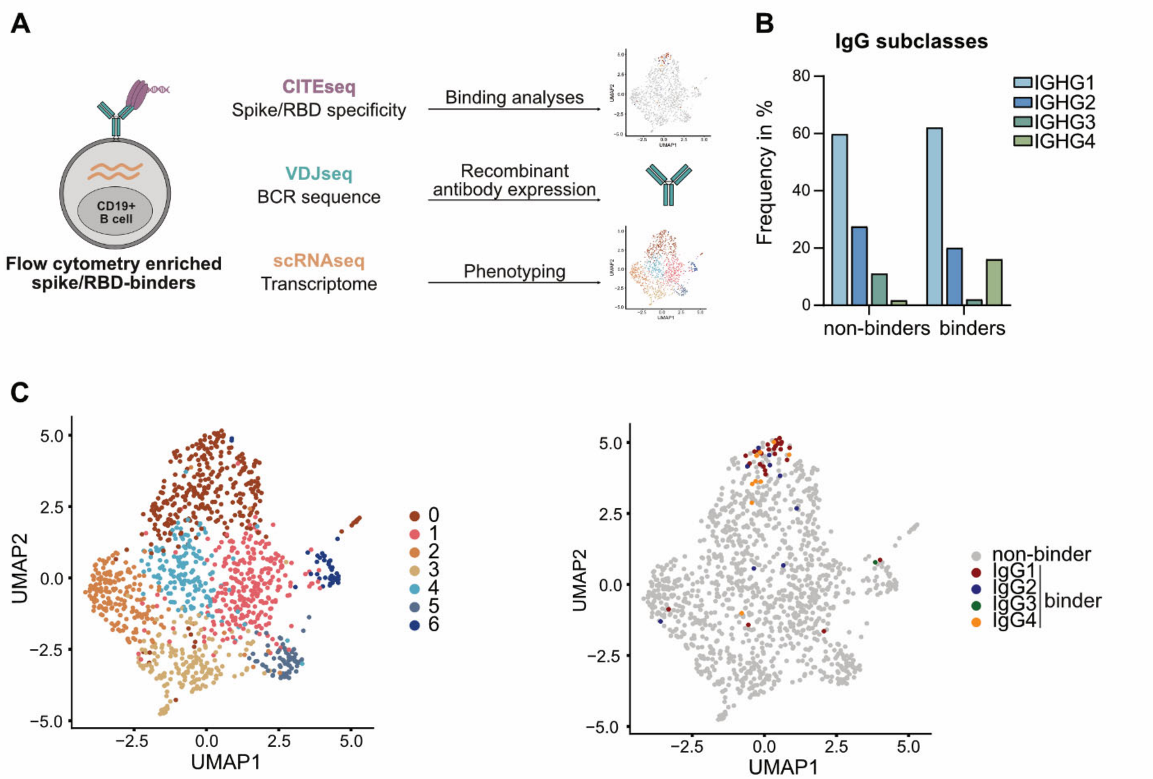

D

Donor	Anti-spike IgG4 210 days after 2nd (µg/ml)	Anti-spike IgG4 10 days after 3rd (µg/ml)	Total number of spike- binding single-cells	Number (frequency) of spike-binding memory B cell			
				γ1	γ2	γ3	γ4
14	1.9	43.9	4	3 (75%)	0	0	1(25%)
15	0.7	2.3	13	9 (69%)	4 (31%)	0	0
22	4.2	143.2	27	17 (63%)	6 (22%)	1 (4%)	3 (11%)
31	3.1	665.5	6	2 (33%)	0	0	4 (67%)

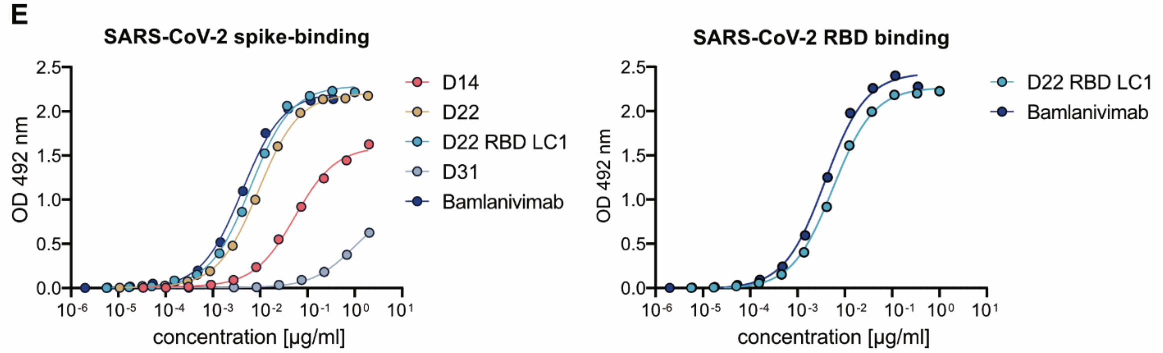

Figure S4: Single-cell RNA, CITE and VDJ sequencing of spike-binding memory B cells

(**A**) Experimental design for scRNA-seq data generation including CITE-seq and BCR-VDJseq. (**B**) Frequency distribution of IgG subclasses obtained from scRNA dataset.

Samples from four individual donors and two time points (210 days post 2nd and 10 days post 3rd vaccination) were analyzed in parallel using hashtag-technology. Aggregate isotype frequencies of spike binders (n=50) and non-binders (n=1004) are shown. (**C**) UMAP visualization of all CD19$^+$ IgG$^+$ sorted cells (1004 non-binders and 50 spike-binders) from all four donors and two time points that passed quality control. The cells are represented in seven main clusters (left). Spike binders were projected to the UMAP visualization with different colors depicting different subclasses (right). (**D**) The table shows the anti-S IgG4 levels and the number of spike-binding memory B-cells for the four selected donors. (**E**) Characteristics of the produced recombinant antibodies derived from scRNA-seq data from mRNA vaccinees. Heavy and light chain sequence information were extracted from four IgG4 spike-binding cells obtained from scRNA-seq. ELISA binding curve to SARS-CoV-2 protein (left) or ELISA binding curve to RBD peptide (right). The therapeutic SARS-CoV-2-specific monoclonal antibody bamlanivimab was used as positive control.

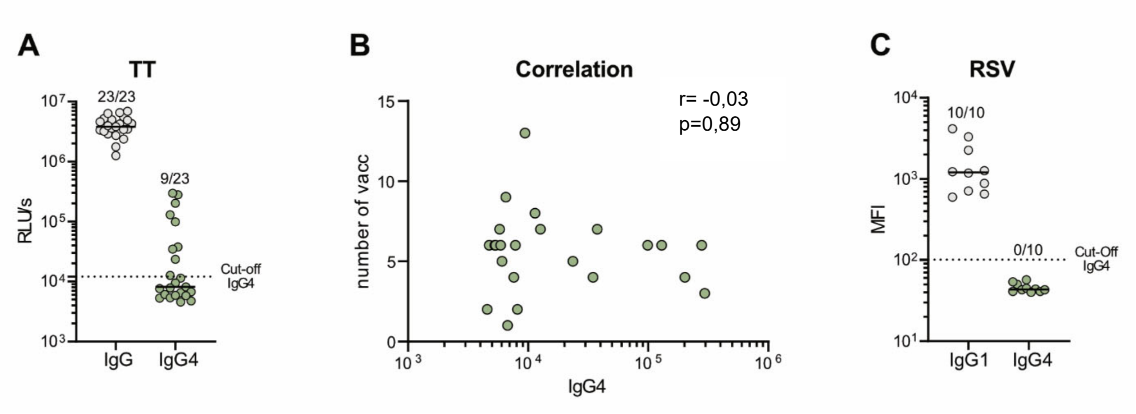

Figure S5: IgG4 antibody response to tetanus vaccinations or RSV infections

(**A**) In 23 individuals with a history of multiple vaccination against tetanus toxoid (see Table S3), TT-specific IgG and IgG4 antibodies were analyzed by ELISA. (**B**) A non-parametric correlation was computed for the individuals IgG4 levels and the number of vaccine doses. The Spearman correlation coefficient and the p-value are shown. (**C**) Using our FACS-based antibody assay with RSV-F protein expressing cells, RSV-specific IgG1 and IgG4 antibodies were measured in ten randomly selected sera of cohort 2. Dots represent individual sera with respective median. Number of positive sera are indicated for each analysis.

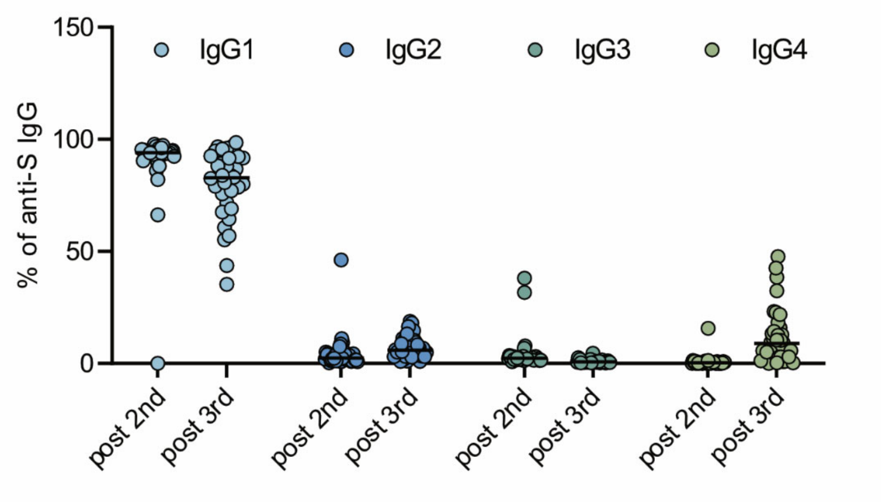

Figure S6: Relative abundance of IgG subclasses in cohort 2

The relative abundance of the different anti-S IgG subclasses is shown for each of the 38 individuals of cohort 2 ten days after second (post 2nd) and third (post 3rd) immunization.

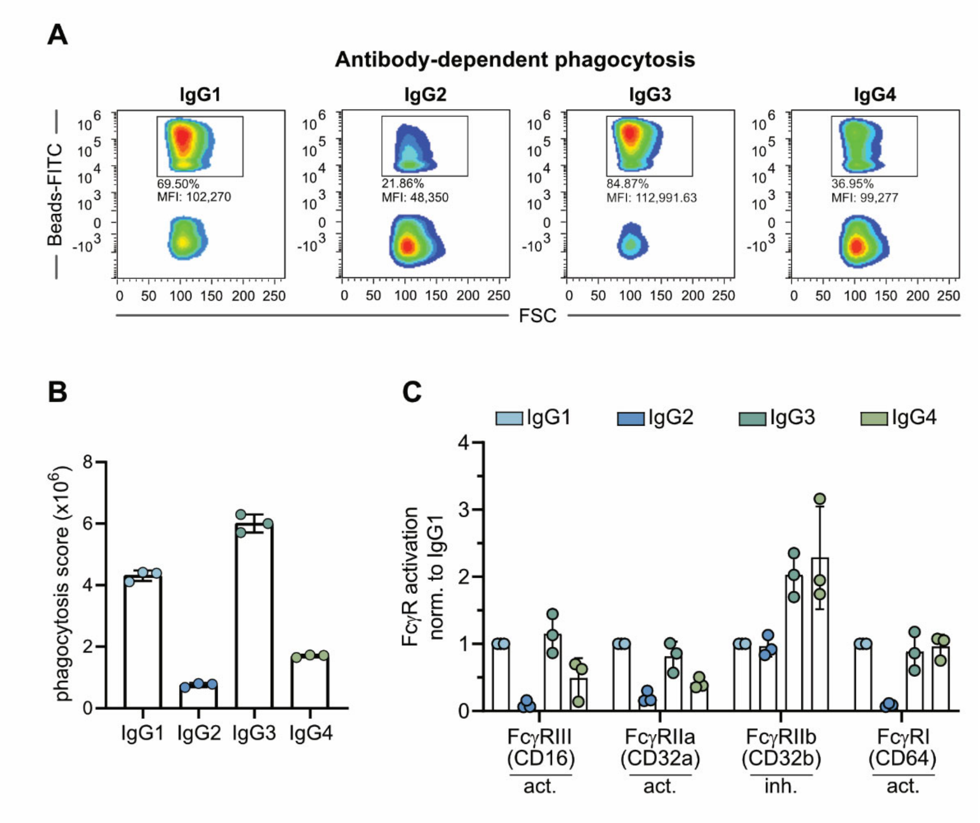

Figure S7: Antibody-dependent phagocytosis and FcγR reporter assays

(**A**) Representative plots for IgG-mediated phagocytosis of spike-loaded microbeads by THP-1 cells are shown for the monoclonal RBD antibodies of the different subclasses (IgG1, IgG2, IgG3, IgG4). (**B**) The phagocytic scores were quantified for each monoclonal antibody. **(C)** Using a panel of FcγR reporter cells, the monoclonal RBD antibodies were analyzed for their potential to activate the different human FcR receptors CD16A, CD32A, CD32B and CD64. The level of IL-2 as a measure of the respective FcγR activation was first normalized to the total amount of spike-binding antibodies. Furthermore, the activation of the different FcγR by the IgG2, IgG3 and IgG4 isotypes were normalized to the respective activation by the IgG1 mAb. Dots represent each independent experiment (n=3).

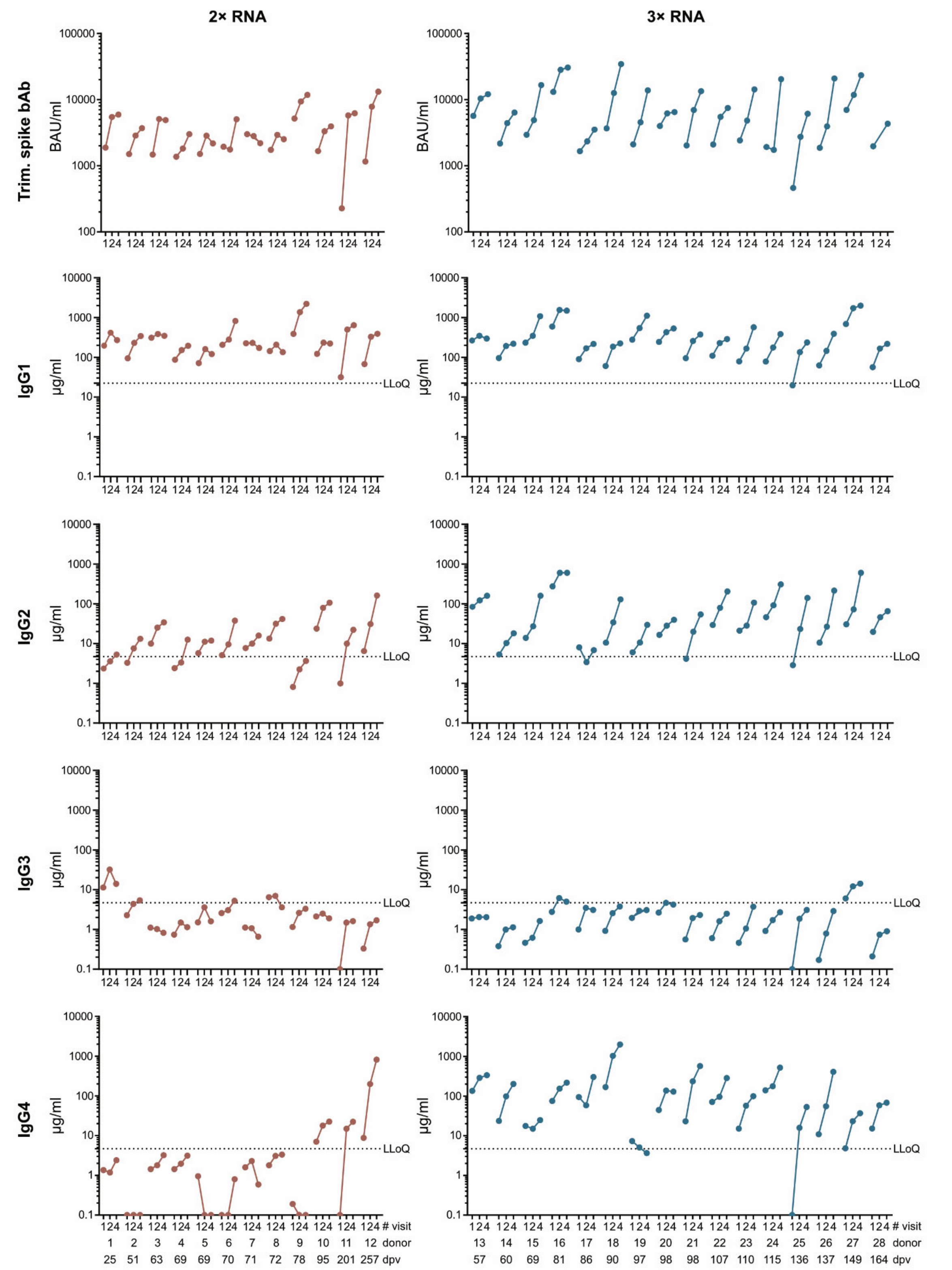

Figure S8: Anamnestic antibody responses after breakthrough infections

In individuals with PCR-confirmed SARS-CoV-2 breakthrough infection (see Table S4), anamnestic antibody responses were quantified at the time point of study inclusion (V1,

typically one week after PCR) as well as two (V2) and four weeks (V4) after the PCR result. Anamnestic responses in individuals experiencing infection after two (left panels) or three (right panels) immunizations were compared. Individuals are ordered from left to right based on increasing time difference between last vaccination and breakthrough infection. Trimeric spike-binding IgG antibodies were analyzed in fully automated CLIA assay (LIAISON®SARS-CoV-2 TrimericS IgG assay). Antibody levels were quantified according to the WHO International Reference standard and listed as BAU/ml. The levels of anti-spike IgG1, IgG2, IgG3 and IgG4 were quantified by flow cytometry. The lowest limit of quantification (LLoQ) is indicated in each graph by a dotted line and represents the lowest detectable amount of the respective standard mAbs (4.68 µg/ml for IgG2, IgG3 and IgG4; 22.4 µg/ml for IgG1). Individual responses were indicated by dots with a connecting line.

Table S1: Characteristics of study cohort comparing homologous vs. heterologous vaccination

	Het-Imm (Erlangen)[37]		
prime<br>boost	BNT162b2<br>BNT162b2	ChAdOx1 nCoV-19<br>ChAdOx1 nCoV-19	ChAdOx1 nCoV-19<br>BNT162b2
Number of volunteers	n = 30	n = 21	n = 30
Age in years, median (IQR) [range]	47 (32-54)<br>[24-60]	45 (40-55)<br>[31-59]	46 (33-54)<br>[21-59]
Sex, n (%) Female	15 (50 %)	15 (71.4 %)	15 (50 %)
Sex, n (%) Male	15 (50 %)	6 (28.6 %)	15 (50 %)
Time intervals between immunizations in days, median (IQR) [range]	23 (22-25)<br>[18-28]	63 (63-63)<br>[63-63]	63 (63-63)<br>[63-63]
Time interval from immunization to blood collection in days, median (IQR) [range]	155 (153-164)<br>[148-177]	142 (141-144)<br>[140-144]	142 (142-144)<br>[141-144]
Overlap with Cohort 2	8	0	0

Table S2: Relative proportion of IgG subclasses among spike and non-spike binding cells switched memory B-cells

Donor	Time point	CD27+ spike^{neg}					CD27+ spike^{pos}					Anti-S IgG4	
		No. of cells	% IgG1	% IgG2	% IgG3	% IgG4	No. of cells	% IgG1	% IgG2	% IgG3	% IgG4	µg/ml	%**
A6	FU 2nd	19676	58.9	27.4	11.2	2.4	521	60.0	15.4	5.7	19.0	1.36	2.9
	post 3rd	15186	62.9	22.6	11.3	3.1	999	77.1	14.0	3.3	5.6	18.25	5.4
	FU 3rd	15368	55.5	30.6	11.4	2.4	568	56.8	27.3	3.1	12.9	1.42	4.1
A9	FU 2nd	11335	57.4	29.3	6.5	6.9	550	74.1	9.8	4.5	11.6	0.2	0.3
	post 3rd	9038	34.5	57.0	6.4	2.1	378	78.2	13.2	3.0	5.6	7.5	2.2
	FU 3rd	8246	62.9	29.3	5.4	2.4	464	64.2	22.0	1.3	12.5	0.21	0.3
A11*	FU 2nd	20668	69.0	20.7	6.7	3.7	433	69.7	23.6	1.2	5.5	2.96	12.3
	post 3rd	28089	79.5	8.1	3.5	8.9	796	68.5	13.7	1.9	15.9	208.72	35.5
	FU 3rd	9279	65.8	23.8	8.6	1.8	614	52.6	27.5	0.8	19.1	170.31	47.8
A13*	FU 2nd	11086	67.7	26.6	4.0	1.7	379	61.4	27.2	0.8	10.6	0.18	0.7
	post 3rd	3265	69.8	22.2	7.1	0.9	493	57.4	30.0	5.9	6.7	16.62	3.6
	FU 3rd	10636	63.4	25.8	9.9	0.9	520	59.8	23.5	2.7	14.0	20.34	3.8
A16	FU 2nd	16868	57.5	29.6	10.7	2.2	356	67.1	19.4	0.6	12.9	0.21	2.6
	post 3rd	19063	58.9	31.0	7.9	2.2	626	65.0	19.8	0.9	14.4	30.9	15.2
	FU 3rd	14097	67.4	19.7	10.0	2.9	461	43.5	41.8	0.4	14.2	16.62	23.7
A17	FU 2nd	15390	79.0	15.5	4.8	0.7	203	69.9	17.7	1.0	11.4	0.13	0.6
	post 3rd	18070	77.9	16.8	4.9	0.3	706	73.3	18.4	0.7	7.6	13.27	1.6
	FU 3rd	10842	58.2	30.0	10.7	1.2	414	55.2	34.3	0.9	9.6	2.25	1.1
A18	FU 2nd	12577	23.0	72.2	3.6	1.2	32	46.5	31.7	0.0	21.9	0.62	3.1
	post 3rd	12897	21.2	75.5	2.7	0.6	119	49.4	31.9	3.6	15.1	5.92	2.7
	FU 3rd	10738	28.5	66.8	3.6	1.1	45	30.9	59.5	1.9	7.7	0.1	0.9
A19	FU 2nd	17186	53.9	26.3	18.0	1.8	252	59.1	20.7	0.4	19.8	1.19	5.7
	post 3rd	28813	52.3	33.2	13.4	1.1	750	57.1	22.7	2.1	18.1	60.11	26.8
	FU 3rd	18797	47.7	37.9	12.9	1.4	297	47.5	22.9	1.8	27.8	14.24	39.4
A24	FU 2nd	34464	40.5	50.1	8.2	1.1	902	71.4	14.7	4.5	9.3	3.33	9.8
	post 3rd	34779	42.8	46.7	9.5	0.9	1634	73.0	14.4	3.2	9.4	19.91	8.9
	FU 3rd	31946	43.9	46.0	9.0	1.1	1206	71.7	14.5	2.2	11.6	0.97	2.4
A28*	FU 2nd	25061	61.7	27.9	8.6	1.8	295	55.4	15.9	2.9	25.7	1.95	8.5
	post 3rd	23794	63.8	24.8	10.1	1.3	1066	24.2	42.6	0.4	32.8	0.1	0.2
	FU 3rd	22215	60.9	27.6	10.2	1.2	606	26.7	35.3	0.7	37.2	634.03	68.4
A29*	FU 2nd	9117	50.8	41.2	6.1	2.0	109	72.7	21.1	2.3	3.9	1.32	5.6
	post 3rd	20902	50.2	39.8	7.8	2.2	661	49.0	23.7	1.2	26.1	95.21	23.7
	FU 3rd	16787	50.2	40.3	7.5	2.0	724	55.3	17.4	0.3	27.0	362.78	49.8

* had breakthrough infection in the time interval between post 3rd and FU 3rd
** percentage of sum of all IgG subclasses

Table S3: Cohort with history of TT vaccinations

Donor	Years since last dose	# of vaccine doses
U001	10	9
U002	7	6
U003	8	7
U004	7	4
U005	1	8
U006	7	5
U007	0.2	2
U008	10	7
U009	6	2
U010	4	13
U011	10	4
U012	6	6
U013	4.5	6
U014	5	1
U015	7	4
U016	4	7
U017	4	6
U018	2	5
U019	1	6
U020	6	6
U021	6	3
U022	2	6
U023	5	6

Table S4: Characteristics of cohort (CoVaKo study) with breakthrough infections after two or three mRNA vaccine immunizations

ID	Sex	Age (years)	Vaccines	VOC	Time span between last immunization and infection (days)
1	female	54	BB	Delta	25
2	female	33	BB	Delta	51
3	female	51	BB	Alpha	63
4	male	31	BB	Delta	69
5	female	31	BB	Delta	69
6	male	28	BB	Omicron BA.2	70
7	male	52	BB	Delta	71
8	male	24	BB	Delta	72
9	female	30	BB	Omicron BA.2	78
10	female	58	BB	Alpha	95
11	female	38	BB	Omicron BA.2	201
12	female	42	BB	Delta	257
13	female	36	BBM	Omicron BA.1	57
14	male	19	BBB	Omicron BA.2	60
15	female	38	BBM	Omicron BA.1	69
16	female	39	BBB	Omicron BA.1	81
17	male	41	BBB	Omicron BA.1	86
18	male	39	BBB	Omicron BA.1	90
19	male	66	BBB	Omicron BA.1	97
20	female	22	BBB	Omicron BA.2	98
21	male	25	BBB	Omicron BA.2	98
22	female	28	BBB	Omicron BA.2	107
23	male	43	BBM	Omicron BA.2	110
24	female	62	BBM	Omicron BA.2	115
25	female	41	BBB	Omicron BA.2	136
26	female	39	BBB	Omicron BA.2	137
27	male	53	BBB	Omicron BA.2	149
28	male	59	BBB	Omicron BA.2	164

B= BioNTech/Pfizer; M= Moderna
